# Teacher-regulated generative AI support, student agency, and perceived learning gains in higher education: the moderating role of perceived fairness

**DOI:** 10.3389/frai.2026.1863666

**Published:** 2026-07-13

**Authors:** Zeyu Zhang, Xiaomei Lu, Mahpiret Kanji

**Affiliations:** 1 Judicial Police College, Xinjiang University of Political Science and Law, Tumxuk, China; 2 School of Educational Science, Xinjiang Normal University, Urumqi, China; 3 Nursing Department, Sihong Hospital, Suqian, China; 4 Xinjiang Key Laboratory of Mental Development and Learning Science, Xinjiang Normal University, Urumqi, China

**Keywords:** Generative AI, higher education, perceived fairness, perceived learning gains, student agency, teacher-regulated generative AI support

## Abstract

**Introduction:**

Generative artificial intelligence is increasingly used in higher education, yet its educational value depends not only on technological access but also on how its use is pedagogically regulated. This study examined how teacher-regulated generative AI support is associated with university students' perceived learning gains in higher education. It further tested whether student agency mediates this association and whether perceived fairness conditions the strength of the association between teacher-regulated generative AI support and student agency.

**Methods:**

We adopted a cross-sectional quantitative survey design and collected data from 428 university students from six universities in western China who had used generative AI in course-related learning tasks under teacher guidance and within teacher-defined expectations. Structural equation modeling was used to test the direct and indirect associations in the proposed model, and a manifest interaction analysis was used to examine the first-stage moderation effect of perceived fairness.

**Results:**

In the proposed model, teacher-regulated generative AI support was positively associated with perceived learning gains both directly and indirectly through student agency. Student agency was statistically consistent with a mediated pathway linking teacher-regulated generative AI support to perceived learning gains. In addition, perceived fairness was associated with a stronger positive relationship between teacher-regulated generative AI support and student agency.

**Discussion:**

These findings suggest that students reported greater learning benefits when generative AI use was guided more clearly by teachers, taken up more actively by learners, and experienced in a fairer learning context. The study therefore highlights student agency as one plausible pathway linking teacher-regulated AI use to perceived learning gains in higher education.

## 1 Introduction

### 1.1 Generative AI in higher education

Generative artificial intelligence is increasingly being integrated into higher education through applications such as tutoring, personalized feedback, adaptive explanations, and interactive learning support (Coman et al., 2026; Dogaru et al., 2025; Niu et al., 2026). At the same time, its use has raised growing concerns about plagiarism, misinformation, academic integrity, and responsible governance in academic settings (Thomson et al., 2024; Coman et al., 2026; Dogaru et al., 2025). Recent research suggests that the educational value of generative AI is not explained by access alone, but by how AI use is pedagogically organized, actively taken up by learners, and embedded in fair learning conditions (Alqurni, 2026; Coman et al., 2026). This makes higher education an especially important context for examining the roles of student agency, perceived fairness, and perceived learning gains in AI-supported learning.

At the same time, the educational significance of generative AI depends not merely on its technical capabilities, but on how it is embedded in real learning environments. In higher education, AI use increasingly intersects with assessment practices, institutional policies, explainability, and students' evolving expectations of academic support (Thomson et al., 2024; Khosravi et al., 2022). As a result, the key issue is no longer simply whether generative AI is available to students, but under what pedagogical and social conditions it supports substantive learning.

### 1.2 From tool access to pedagogical regulation

However, the educational value of generative AI cannot be reduced to technological access alone. Existing research increasingly suggests that learning in AI-supported contexts depends on more than whether students use AI tools; it also depends on how such use is pedagogically organized and institutionally guided (Coman et al., 2026; Qian, 2025; Chiu, 2023). In many higher education contexts, generative AI appears attractive because it can help bridge the gap between the support students seek and what instructors can realistically provide at scale (Thomson et al., 2024; Chan and Tsi, 2024; Niu et al., 2026). Yet AI-supported learning may remain shallow when students simply receive outputs without sufficient instructional framing, critical engagement, or revision-oriented guidance (Kasneci et al., 2023; Lee et al., 2024). Existing research has described the opportunities and risks of AI use, but has less clearly specified how pedagogical regulation may shape students' learning processes and perceived gains. The key issue is not merely whether generative AI is available, but under what pedagogical conditions it becomes pedagogically useful.

This concern shifts attention from tool availability to pedagogical regulation (Wood et al., 1976; van de Pol et al., 2010). In the present study, teacher-regulated generative AI support is defined as students' perceptions that teachers pedagogically regulate the educational use of generative AI by clarifying its goals, acceptable-use boundaries, interpretive expectations, and revision-oriented use in course-related learning tasks. We do not use this term to refer to teacher support in a broad interpersonal sense, generic instructional scaffolding across all learning contexts, autonomy support in the motivational sense, or institution-level AI policy clarity. Rather, the construct is used here as a context-specific operationalization of pedagogical regulation in generative AI-supported higher education. Recent work suggests that teachers remain central in shaping how AI is educationally used, interpreted, and bounded, rather than being displaced by it (Chan and Tsi, 2024). Similarly, research on AI-augmented teaching models indicates that AI becomes more pedagogically useful when it is embedded in a coherent instructional framework rather than treated as a stand-alone technical add-on (Niu et al., 2026). Generative AI is better understood as a resource whose value depends on pedagogical mediation, not as an autonomous educational solution.

### 1.3 Student agency and perceived fairness as missing explanatory links

A second unresolved issue concerns the learner processes through which pedagogically regulated AI support may relate to learning outcomes. Prior research has shown growing interest in agency, self-regulated learning, autonomy support, self-efficacy, and self-learning motivation in AI-assisted contexts, suggesting that learning in AI-supported contexts depends on more than technology use; it also depends on how actively students plan, monitor, evaluate, and revise their learning (Alqurni, 2026; Xia et al., 2025; Ryan and Deci, 2020; Zimmerman, 2000). Student agency is especially relevant in this regard because generative AI can either strengthen or weaken students' active role in learning depending on how it is introduced and used. When students critically interpret AI outputs, make purposeful decisions, and revise their work on the basis of their own judgment, AI use is more likely to support substantive learning instead of passive dependence.

In addition, recent scholarship increasingly highlights that the educational effects of generative AI are inseparable from questions of fairness, transparency, policy clarity, and ethical governance. Students' perceptions of whether AI-supported learning is managed fairly may shape how they interpret and respond to teacher-guided AI use. This issue is particularly salient because students often report uncertainty about AI-related rules and institutional expectations, while broader reviews emphasize equity, bias, and responsible governance as central to the future of AI in higher education (García-López and Trujillo-Liñán, 2025; Thomson et al., 2024; Coman et al., 2026). Yet relatively little empirical research has examined whether teacher-regulated generative AI support relates to perceived learning gains through student agency and under what fairness conditions this relationship becomes stronger. In the present study, perceived fairness is treated as a procedural perception of transparency, consistency, and legitimacy in AI-supported learning arrangements rather than as a broad measure of institutional trust or general course satisfaction. Together, these gaps point to the need for an empirical model that explains how teacher-regulated generative AI support relates to perceived learning gains through student agency and how this process varies under different fairness conditions.

### 1.4 Research purpose and contribution

Against this background, the present study examines whether teacher-regulated generative AI support is associated with university students' perceived learning gains in higher education, whether student agency mediates this association, and whether perceived fairness strengthens the positive association between teacher-regulated generative AI support and student agency. We do not claim that prior studies have ignored AI-supported pedagogy, AI literacy, instructional design, AI governance, or instructor roles. Rather, the gap addressed here is more specific and incremental: these strands of research have not yet been sufficiently integrated to explain how teacher-regulated generative AI support, learner agency, and fairness perceptions jointly relate to students' perceived learning gains in authentic course-related AI use. The study therefore makes three contributions. First, it conceptualizes generative AI as a pedagogically regulated learning support arrangement rather than an autonomous technological input embedded in classroom expectations. Second, it identifies student agency as a plausible explanatory pathway through which teacher-regulated generative AI support may be associated with perceived learning gains. Third, it introduces perceived fairness as a contextual boundary condition, thereby extending current discussions of responsible and human-centered AI integration in higher education (Coman et al., 2026; Le Dinh et al., 2025). By linking pedagogical regulation, learner agency, and fairness in a single empirical model, the study clarifies one relational pathway through which teacher-regulated generative AI support may be associated with perceived learning gains in higher education.

## 2 Theoretical background and hypothesis development

### 2.1 Teacher-regulated generative AI support and perceived learning gains

Generative AI has introduced new possibilities for higher education by providing students with rapid feedback, alternative explanations, language support, and task-oriented scaffolding. However, the educational value of such support depends not only on the technical capacity of AI tools, but also on how their use is pedagogically framed in real learning environments (Kasneci et al., 2023; Coman et al., 2026). In this study, teacher-regulated generative AI support is conceptualized as a context-specific form of pedagogical regulation in which teachers shape students' educational use of generative AI through explicit goal framing, acceptable-use boundaries, interpretive guidance, and revision-oriented expectations. This construct is therefore narrower than general teacher support, more AI-specific than generic instructional scaffolding, and more course-level and practice-oriented than broad institutional AI policy clarity. This framing is consistent with research emphasizing that teachers remain central to responsible AI use in higher education and that AI becomes pedagogically useful when embedded in teaching design rather than treated as simple technical convenience (Chan and Tsi, 2024; Niu et al., 2026).

This framing is important because students do not encounter generative AI in a pedagogical vacuum. In higher education, AI tools may be used for idea generation, writing support, reading assistance, concept clarification, and task planning, yet these uses vary considerably in quality and educational value. When AI use is weakly regulated, students may rely on it merely to speed up task completion, reduce effort, or bypass difficult thinking processes (Kasneci et al., 2023; Lee et al., 2024; Michel-Villarreal et al., 2023). By contrast, when teachers explicitly frame AI as a learning support rather than a shortcut, students are more likely to engage with it in ways aligned with course goals, disciplinary expectations, and meaningful revision practices. These differences in the pedagogical framing of AI use are likely to shape whether students experience AI-supported learning as merely efficient or as genuinely beneficial to their academic progress.

Teacher-regulated generative AI support may enhance perceived learning gains in at least three ways. First, it can improve cognitive clarity by helping students understand what AI should and should not be used for in a given learning context. Second, it can increase the instructional value of AI feedback by encouraging students to interpret, question, and revise rather than merely accept generated outputs. Third, it can strengthen the perceived legitimacy of AI-supported learning by embedding AI use within a clear pedagogical structure. Under such conditions, students are more likely to perceive AI as contributing to perceived academic progress rather than only procedural convenience. Research on students' perceptions of AI in higher education likewise suggests that students value AI more when it helps them solve academic problems, clarify understanding, and receive timely support within substantive learning tasks (Dogaru et al., 2025; Thomson et al., 2024).

Perceived learning gains in this study refer to students' self-reported sense of improvement in understanding, performance, efficiency, and perceived academic progress within AI-supported learning experiences. This outcome reflects perceived rather than objectively verified learning improvement. Because teacher-regulated generative AI support provides clearer pedagogical direction and more educationally productive conditions for AI use, it is expected to be positively associated with perceived learning gains.

**H1**. Teacher-regulated generative AI support is positively associated with perceived learning gains.

### 2.2 Teacher-regulated generative AI support and student agency

Student agency refers to learners' active capacity to make purposeful decisions, monitor their understanding, evaluate alternatives, and shape the direction of their own learning. In higher education, student agency is central to substantive learning because university students are expected not merely to receive information, but to interpret, critique, and use it in self-directed ways. In AI-supported contexts, this issue becomes especially important because generative AI can either strengthen or weaken students' active role in learning depending on how it is introduced and used. Recent studies of agentic AI, autonomy support, and AI-supported self-learning emphasize that productive AI-assisted learning depends on system capability as well as the extent to which learners remain active, self-directing, and confident in shaping their own learning (Alqurni, 2026; Xia et al., 2025). More broadly, scholarship on agentic engagement suggests that students learn more effectively when they actively contribute to the regulation of their own learning environment rather than merely respond to external inputs (Reeve, 2013).

Teacher-regulated generative AI support may strengthen student agency by positioning students as active decision-makers rather than passive recipients of machine-generated output. When teachers clarify why and how AI should be used, encourage students to examine the quality of AI-generated responses, and require them to justify their revisions or learning choices, students are pushed to exercise judgment rather than merely consume answers. Seen this way, teacher guidance may shift AI use away from quick answer-getting and toward a more deliberate form of learning in which students still need to judge, revise, and take responsibility for their work.

This argument is plausible because guidance does not always reduce autonomy. In many learning situations, clearer structure can actually help students act more purposefully (Ryan and Deci, 2020; Reeve, 2013). Instructional guidance may therefore function as a form of autonomy-supportive scaffolding by reducing ambiguity, clarifying expectations, and enabling more purposeful learner action (Ryan and Deci, 2020; van de Pol et al., 2010). Students are more likely to act agentically when they understand the goals of the task, the role of AI within it, and the standards by which their work will be judged. Thus, teacher regulation may serve as a scaffold that enables agency rather than suppresses it.

In the present study, student agency is reflected in such behaviors as deciding how to use generative AI, critically evaluating its suggestions, revising work on the basis of one's own judgment, and maintaining ownership of the learning process. Because teacher-regulated generative AI support creates more structured and purposeful conditions for AI-assisted learning, it is expected to be positively associated with student agency.

**H2**. Teacher-regulated generative AI support is positively associated with student agency.

### 2.3 Student agency and perceived learning gains

Student agency is expected to be positively associated with perceived learning gains because substantive learning depends on both the availability of support and the way learners actively work with that support. Students who plan their learning, monitor their understanding, evaluate information critically, and revise their work deliberately are more likely to translate external assistance into deeper understanding and sustained academic progress. In higher education, learning gains are therefore shaped not only by instructional resources, but also by the extent to which students experience themselves as active participants in knowledge construction. This proposition aligns with research from social cognitive theory and self-determination theory, both of which suggest that agency, motivation, autonomy, and perceived competence are closely tied to more positive learning outcomes (Bandura, 1986; Ryan and Deci, 2020; Howard et al., 2021).

This logic is especially relevant in generative AI-supported learning. Although AI tools can provide rapid answers, summaries, examples, and suggestions, they do not by themselves guarantee substantive learning. Educational value depends on whether students verify AI-generated information, adapt it to task requirements, identify its limitations, and integrate it into their own thinking. When students use AI in these active ways, the support is more likely to be linked to genuine understanding and improvement. When students rely on AI passively, by contrast, the convenience of the tool may not translate into academic development. Studies on students' use of generative AI in academic learning likewise suggest that active engagement with AI is associated with stronger learning retention, competence, and meaningful use, whereas superficial reliance may increase dependence and weaken critical engagement (Dogaru et al., 2025; Lee et al., 2024).

The emphasis on student agency is therefore important because it helps explain why the same AI tool may produce different learning experiences for different students. Students with stronger agency are more likely to engage in critical selection, purposeful revision, and reflective use of AI-supported information. These agentic behaviors may enhance their perceived gains in understanding, efficiency, and work quality. Accordingly, student agency is expected to be positively associated with perceived learning gains.

**H3**. Student agency is positively associated with perceived learning gains.

### 2.4 The mediating role of student agency

Taken together, the above arguments suggest that student agency may serve as one plausible explanatory pathway linking teacher-regulated generative AI support to perceived learning gains. Teacher-regulated support may be associated with perceived learning not only because it provides students with clearer pedagogical guidance, but also because it may encourage them to engage more actively with AI-supported tasks. In other words, pedagogical regulation may create the conditions under which students use AI more deliberately, critically, and reflectively, and these agentic learning processes may in turn be associated with stronger perceived gains. This mediation logic is consistent with recent empirical work suggesting that AI-supported learning outcomes are partly explained through self-efficacy, self-regulated learning, and active learner engagement rather than AI exposure alone (Jin et al., 2025; Alqurni, 2026).

This mediation logic is important because it clarifies how AI-supported pedagogy may acquire educational value in the proposed model. It is not sufficient to assume that teacher guidance directly improves learning simply by making AI use more orderly. Rather, the educational value of such guidance may lie partly in the way it supports students' ownership of the learning process. When students understand the purpose of AI use, evaluate outputs rather than accept them uncritically, and revise work using their own judgment, they are more likely to perceive AI-supported learning as beneficial.

Thus, student agency offers a process-based explanation for the relationship between teacher-regulated generative AI support and perceived learning gains. This perspective is consistent with a human-centered view of generative AI in higher education, according to which AI may acquire educational value beyond access and efficiency when learners remain actively involved in meaning-making and decision-making. Accordingly, student agency is expected to mediate the relationship between teacher-regulated generative AI support and perceived learning gains in the proposed model.

**H4**. Student agency mediates the relationship between teacher-regulated generative AI support and perceived learning gains.

### 2.5 The moderating role of perceived fairness

Although teacher-regulated generative AI support may generally strengthen student agency, its effectiveness may depend on how students perceive the fairness of the AI-supported learning environment. In the present study, perceived fairness refers to students' judgments about whether AI-supported learning opportunities are equitable, whether rules governing AI use are transparent, and whether evaluation standards are applied consistently. This concept is particularly relevant in higher education because the introduction of generative AI often raises concerns about unequal access, unclear expectations, inconsistent teacher responses, and ambiguity regarding acceptable use. Recent work on AI governance in education has emphasized the importance of ethical safeguards, transparency, and fair implementation in addition to pedagogical innovation (García-López and Trujillo-Liñán, 2025; UNESCO, 2023; Coman et al., 2026).

Perceived fairness may shape how students interpret pedagogical regulation. When students perceive the AI-supported learning environment as fair, teacher guidance is more likely to be interpreted as legitimate, transparent, and supportive. Under such conditions, students may be more willing to respond to teacher-regulated AI support with active engagement, because they believe that the rules are clear, the opportunities are equitable, and the evaluative context is trustworthy (UNESCO, 2023; García-López and Trujillo-Liñán, 2025; Thomson et al., 2024). In contrast, when students perceive low fairness, even well-intentioned pedagogical guidance may be less effective because students may interpret it as inconsistent, restrictive, or unequally applied. Here, perceived fairness in the present study is not limited to broad governance concerns, but refers more specifically to students' sense that AI-supported learning arrangements are procedurally legitimate, transparent, and consistently implemented (Colquitt, 2001).

This moderating role is theoretically important because it introduces a contextual boundary condition into the relationship between pedagogical regulation and agency. Teacher regulation may not operate in the same way across all learning environments. Its effectiveness may depend partly on whether students feel that the surrounding conditions of AI-supported learning are fair. This is why perceived fairness is theorized to moderate the regulation–agency link specifically: fairness is most directly relevant to whether pedagogical guidance is interpreted as legitimate, transparent, and worth actively taking up. Here, fairness is not simply an ethical issue external to learning; it may also shape the extent to which students become active participants in AI-mediated educational processes (Coman et al., 2026; UNESCO, 2023).

Accordingly, perceived fairness is expected to strengthen the positive association between teacher-regulated generative AI support and student agency, such that the relationship is stronger when perceived fairness is higher.

**H5**. Perceived fairness positively moderates the relationship between teacher-regulated generative AI support and student agency, such that the relationship is stronger when perceived fairness is higher.

### 2.6 Conceptual framework and hypotheses

The proposed model is also informed by selected Information Systems perspectives. TAM and UTAUT suggest that technology-enabled practices are shaped not only by access, but also by perceived usefulness, ease of use, social influence, and facilitating conditions (Davis, 1989; Venkatesh et al., 2003). In the present study, teacher-regulated generative AI support is conceptually related to facilitating conditions because it provides students with task-level guidance, interpretive expectations, and acceptable-use boundaries for using generative AI in course-related learning. At the same time, technology-adoption models alone do not fully specify how teacher guidance becomes pedagogically useful within learning processes. The present framework therefore complements IS adoption perspectives by focusing on student agency as an active learner process and perceived fairness as a legitimacy condition in AI-supported learning environments.

Based on the above theoretical reasoning, this study proposes a model in which teacher-regulated generative AI support is associated with perceived learning gains directly and indirectly through student agency, while perceived fairness moderates the relationship between teacher-regulated generative AI support and student agency. The model reflects a socio-technical understanding of generative AI in higher education: AI tools gain educational value when they are embedded in instructional practices, taken up actively by learners, and experienced within learning conditions perceived as fair and legitimate (Orlikowski, 2000; Shneiderman, 2022; Le Dinh et al., 2025).

More specifically, the model assumes that teacher-regulated generative AI support provides structured pedagogical conditions for meaningful AI use, that student agency serves as a key explanatory pathway through which such support relates to perceived learning gains, and that perceived fairness conditions the strength of the link between pedagogical regulation and learner agency. In this way, the model integrates pedagogical structure, learner process, and contextual condition into one explanatory framework.

**Figure 1:**
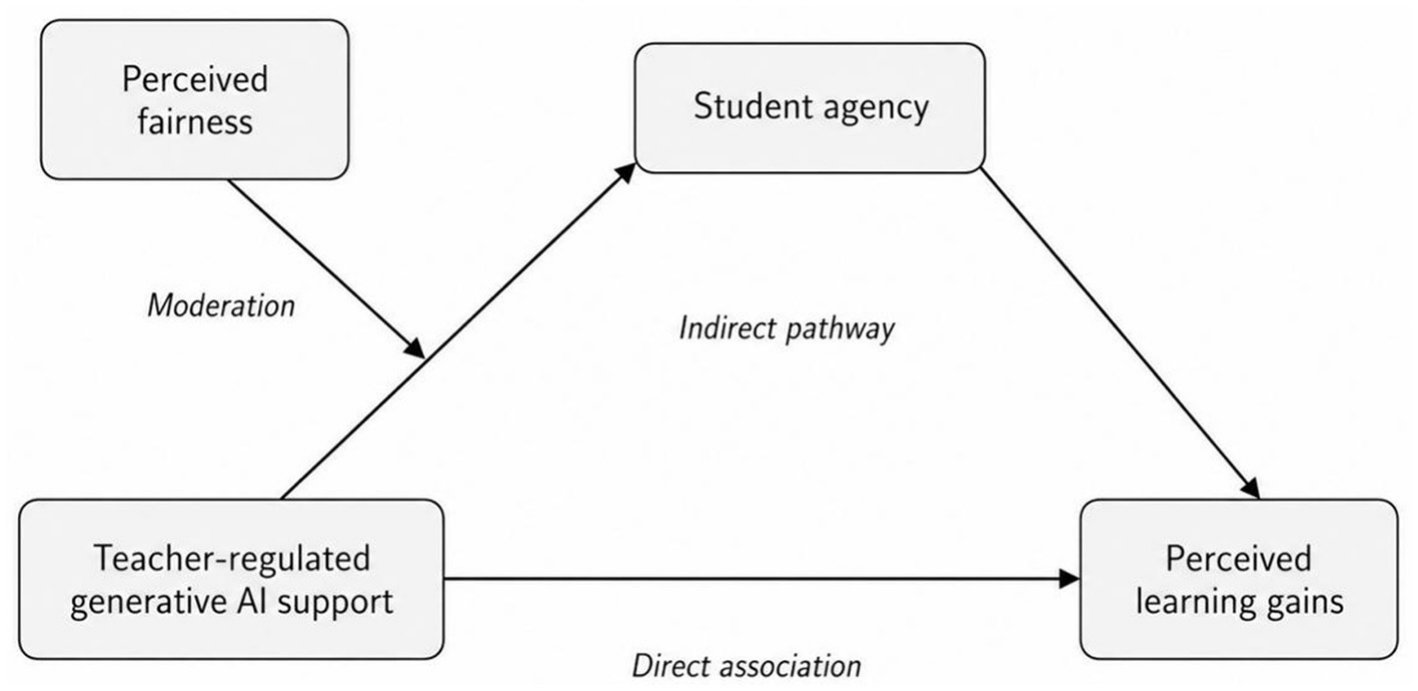
Proposed conceptual framework of teacher-regulated generative AI support, student agency, perceived fairness, and perceived learning gains. Arrows indicate theoretically specified associations. Perceived fairness is modeled as a first-stage moderator of the association between teacher-regulated generative AI support and student agency.

Alternative model specifications are possible. Perceived fairness could be modeled as a direct antecedent of perceived learning gains, and student agency could also shape how students evaluate teacher regulation. However, given the present focus on teacher-guided AI use in authentic course-related learning tasks, the proposed model is theoretically appropriate because teacher regulation is expected to provide the pedagogical conditions under which students actively engage with AI-supported tasks, while fairness shapes whether such regulation is interpreted as legitimate and worth taking up. The model should therefore be interpreted as a theoretically specified relational model rather than a confirmed causal sequence. Figure 1 summarizes the proposed conceptual framework.

## 3 Methods

### 3.1 Participants and procedure

This study employed a cross-sectional survey design to examine how teacher-regulated generative AI support relates to university students' perceived learning gains in higher education. Participants were undergraduate and postgraduate students from six universities in western China who had prior experience using generative AI in course-related learning tasks. Data were collected from October 15, 2025 to December 20, 2025 through an anonymous online questionnaire administered via Wenjuanxing and distributed through university course groups, class WeChat/QQ groups, and learning-management-system notices. Because distribution relied on accessible course and communication channels rather than probability-based sampling, the final sample should be understood as a convenience sample. The participating institutions were accessed through convenience-based recruitment via existing teaching and communication channels.

The six institutions included comprehensive, normal, and application-oriented universities, allowing variation in academic context while remaining within the same broad regional setting. This helped ensure that the proposed model was examined across students with different disciplinary backgrounds and learning environments. The study focused on recalled teacher-guided uses of generative AI in authentic coursework rather than on a single standardized course or platform, and the resulting learning episodes may therefore have varied across participants.

To ensure that responses were grounded in authentic educational contexts, participants were included only if they reported prior use of generative AI in a teacher-guided or teacher-regulated academic context. At the beginning of the survey, participants were asked to recall a recent course or learning task in which they had used generative AI under teacher guidance or within teacher-defined expectations. They were then instructed to answer all subsequent items with reference to that specific learning experience. This procedure was used to anchor responses in a concrete and comparable AI-supported learning episode rather than in students' general attitudes toward generative AI. At the same time, because recalled experiences may have involved different courses, tasks, and AI tools, the design also introduces heterogeneity and possible recall bias that should be considered when interpreting the findings.

Before completing the questionnaire, participants were informed of the purpose of the study, the voluntary nature of participation, the confidentiality of their responses, and their right to withdraw at any time without penalty. Only those who provided informed consent were allowed to proceed to the survey. To improve response quality, the questionnaire included two attention-check items and a contextual screening item designed to confirm relevant AI-supported learning experience.

A total of 467 responses were received. Responses were excluded if they met one or more of the following criteria: (a) more than 10% missing responses, (b) failure on either of the two attention-check items, (c) straight-lining across the core construct items, or (d) a completion time below one-third of the median completion time. After data screening, the final analytical sample consisted of 428 valid responses.

Among the final sample, 42.3% were male, 55.8% were female, and 1.9% preferred not to disclose their gender. Most participants were undergraduates (72.9%), while 27.1% were postgraduates. In terms of disciplinary background, 41.1% were from the humanities and social sciences, 37.9% were from science and engineering, and 21.0% were from other disciplines. Regarding the frequency of generative AI use for academic purposes, 14.3% reported rare use, 34.1% reported occasional use, 35.3% reported frequent use, and 16.4% reported very frequent use.

### 3.2 Measures

All focal constructs were measured using multi-item scales adapted to the context of teacher-guided generative AI use in higher education. Unless otherwise stated, all items were rated on a five-point Likert scale ranging from 1 (strongly disagree) to 5 (strongly agree), with higher scores indicating higher levels of the corresponding construct. Supplementary Table S1 summarizes construct sources, adaptation logic, and development status, and Supplementary Table S2 reports the full item wording. The questionnaire was administered in Chinese. Because the items were developed, adapted, and finalized directly for Chinese-speaking participants in the present study, a separate back-translation procedure was not applicable.

The initial item pool was developed from prior literature on pedagogical support in AI-assisted learning, student agency in technology-supported learning, fairness and governance in educational AI, and perceived learning gains in academic settings (Xia et al., 2025; Thomson et al., 2024; García-López and Trujillo-Liñán, 2025). The draft questionnaire was reviewed by three experts in educational technology and higher education, revised for wording precision and contextual fit, and then piloted with 40 students who were not included in the final analytical sample. The expert review focused on construct relevance, wording clarity, contextual appropriateness, and item redundancy. No item was removed solely on the basis of the pilot test, but minor wording refinements were made before final administration. Details of construct sources, adaptation logic, item-development status, and full item wording are reported in Supplementary Tables S1, S2.

Because the two context-specific constructs were theory-informed and specified *a priori*, the present study used confirmatory factor analysis (CFA) rather than EFA to evaluate the hypothesized measurement structure. A formal Content Validity Index was not calculated; instead, content validity was addressed through literature-based item development, expert review, and pilot testing. The measures should therefore be interpreted as context-specific operationalizations that require further validation in future studies, rather than as fully standardized scales.

#### 3.2.1 Teacher-regulated generative AI support

Teacher-regulated generative AI support was defined as students' perceptions that teachers provided pedagogical regulation for the educational use of generative AI in course-related learning tasks. In the present study, this construct was treated as a context-specific operationalization of pedagogical regulation in generative AI-supported learning rather than as a broad measure of teacher support, generic instructional scaffolding, autonomy support, or institution-level AI policy clarity. It emphasizes the extent to which teachers framed AI use through explicit goals, acceptable-use boundaries, interpretive guidance, and revision-oriented expectations.

Representative items included: “Teachers clearly explained how generative AI should be used in course learning,” “Teachers provided guidance on how to evaluate or interpret AI-generated content,” “Teachers set clear expectations for revising or improving work after using generative AI,” and “Teachers helped students use generative AI in ways that supported learning rather than simple task completion.” These items were newly composed from prior conceptual literature on teacher roles in generative AI-supported higher education and AI-augmented teaching design (Chan and Tsi, 2024; Niu et al., 2026). Higher scores reflected stronger perceived pedagogical regulation of generative AI use by teachers.

#### 3.2.2 Student agency

Student agency referred to students' active role in directing, monitoring, and shaping their learning during AI-supported academic tasks. In the present study, the construct focused on purposeful planning, critical judgment, active revision, and self-directed decision-making in the use of generative AI. It was intended to capture active learner ownership in AI-supported tasks rather than general motivation or self-efficacy.

Representative items included: “I actively decided how to use generative AI to support my learning,” “I evaluated whether AI-generated suggestions were appropriate for my task,” “I revised my work based on my own judgment rather than relying entirely on AI output,” and “I used generative AI in ways that helped me take greater control of my learning process.” Higher scores indicated stronger student agency in AI-supported learning.

#### 3.2.3 Perceived fairness

Perceived fairness was defined as students' perceptions that AI-supported learning opportunities, rules, and evaluation standards were transparent, equitable, and consistently applied (Colquitt, 2001). This construct captures whether students experienced the AI-supported learning environment as procedurally legitimate and just. It was not intended as a broad indicator of institutional trust or general course satisfaction.

Representative items included: “The rules for using generative AI in this course were clear and transparent,” “Students had fair opportunities to benefit from generative AI support,” “The teacher applied similar standards when evaluating AI-supported work,” and “I felt that the use of generative AI in this learning context was managed fairly.” Higher scores indicated stronger perceptions of fairness.

#### 3.2.4 Perceived learning gains

Perceived learning gains referred to students' self-reported sense of improvement in understanding, performance, efficiency, and perceived academic progress through AI-supported learning experiences. This construct captures students' experienced sense of learning benefit rather than objective performance outcomes.

Representative items included: “Using generative AI in this course helped me better understand the learning content,” “Generative AI support helped me improve the quality of my academic work,” “I learned more efficiently when generative AI was used under teacher guidance,” and “My use of generative AI in this learning context contributed to meaningful learning progress.” Higher scores indicated stronger perceived learning gains.

### 3.3 Control variables

Several background variables were included as controls because they may be associated with students' AI-supported learning experiences and perceived academic benefits (Thomson et al., 2024; Wang et al., 2024). These variables included gender, academic level, disciplinary area, and frequency of generative AI use for academic purposes. In the structural analyses, these controls were specified as predictors of student agency and perceived learning gains.

Gender was included because prior educational technology research suggests that technology-related perceptions and learning experiences may vary across student groups. Academic level was controlled because undergraduate and postgraduate students may differ in learning autonomy, task demands, and AI use patterns. Disciplinary area was included because AI use may differ across academic fields with different epistemic and task structures. Finally, frequency of generative AI use was controlled because students with greater prior exposure to AI tools may report different perceptions of support, agency, fairness, and learning gains. The focal structural relations were interpreted after adjustment for these controls.

### 3.4 Data analysis

The data analysis proceeded in four stages. Because the study relied on cross-sectional self-report survey data, the proposed model was tested as a theoretically informed relational model rather than a causal model.

First, descriptive statistics and bivariate correlations were computed for all study variables. Means, standard deviations, and Pearson correlation coefficients were used to examine the overall distribution of the data and the preliminary relationships among the focal constructs.

Second, confirmatory factor analysis (CFA) was conducted to assess the adequacy of the four-factor measurement model. Internal consistency reliability was evaluated using Cronbach's alpha and composite reliability (CR), while convergent validity was assessed through average variance extracted (AVE), following conventional psychometric recommendations (Nunnally and Bernstein, 1994). Discriminant validity was examined by comparing the square roots of AVE with the inter-construct correlations (Fornell and Larcker, 1981). Model fit was evaluated using χ^2^/df, CFI, TLI, RMSEA, and SRMR. In addition, HTMT values were calculated to further assess discriminant validity (Henseler et al., 2015).

Third, structural equation modeling (SEM) was used to test the hypothesized relationships among teacher-regulated generative AI support, student agency, perceived fairness, and perceived learning gains. The direct effects specified in H1–H3 and the indirect pathway proposed in H4 were examined within the structural model. The indirect effect was estimated using bias-corrected bootstrapping with 5,000 resamples and 95% confidence intervals. To evaluate whether the indirect pathway varied across levels of perceived fairness, conditional indirect effects and the index of moderated mediation were also examined (Hayes, 2015). Control variables were included as predictors of student agency and perceived learning gains in the structural model.

Fourth, the moderating effect of perceived fairness proposed in H5 was tested using a manifest interaction approach. Composite mean scores were computed for the focal constructs, and the interaction term between teacher-regulated generative AI support and perceived fairness was created after mean-centering the constituent variables. This approach was selected for analytical transparency and interpretability in the context of the present survey design, although it is less rigorous than latent interaction modeling and should therefore be interpreted with appropriate caution. Simple slope analyses were conducted at high and low levels of perceived fairness to illustrate the interaction pattern.

Descriptive statistics, reliability analyses, and bivariate correlations were conducted in SPSS 26.0, whereas confirmatory factor analysis, structural equation modeling, bootstrapping, and interaction analyses were performed in Mplus 8.3. All CFA and SEM analyses were estimated using robust maximum likelihood (MLR). Because the focal indicators were measured on five-point Likert scales and showed acceptable distributions, they were treated as continuous indicators. Missing data were handled using full information maximum likelihood (FIML), after excluding cases with substantial missingness during the data-screening stage.

### 3.5 Ethical considerations

This study was approved by the institutional research ethics committee of Xinjiang University of Political Science and Law (Approval No. XJPL-KYC-2025-003). Participation was voluntary and based on informed consent. No personally identifiable information was collected, and all responses were analyzed anonymously.

## 4 Results

### 4.1 Participant characteristics

Table 1 presents the participant characteristics of the final analytical sample. A total of 428 university students were included in the final analytical sample. Among them, 42.3% were male, 55.8% were female, and 1.9% preferred not to disclose their gender. Most participants were undergraduates (72.9%), while 27.1% were postgraduates. In terms of disciplinary background, 41.1% were from the humanities and social sciences, 37.9% were from science and engineering, and 21.0% were from other disciplines. Regarding the frequency of generative AI use for academic purposes, 14.3% reported rare use, 34.1% reported occasional use, 35.3% reported frequent use, and 16.4% reported very frequent use.

**Table 1:** Participant characteristics.

Variable	Category	*n*	%
Gender	Male	181	42.3
	Female	239	55.8
	Prefer not to say	8	1.9
Academic level	Undergraduate	312	72.9
	Postgraduate	116	27.1
Discipline	Humanities and social sciences	176	41.1
	Science and engineering	162	37.9
	Other	90	21.0
Frequency of generative AI use for academic purposes	Rarely	61	14.3
	Occasionally	146	34.1
	Frequently	151	35.3
	Very frequently	70	16.4

Percentages may not total 100 exactly because of rounding.

Overall, the sample reflected variation in academic background and prior experience with generative AI in higher education, providing an appropriate basis for testing the proposed model.

### 4.2 Descriptive statistics, reliability, and correlations

Table 2 reports the descriptive statistics, reliability coefficients, and correlations among the study variables. The mean scores of the four focal variables ranged from 3.58 to 3.81, indicating moderate-to-high levels of teacher-regulated generative AI support, student agency, perceived fairness, and perceived learning gains. The standard deviations ranged from 0.66 to 0.73, suggesting acceptable variability in the responses.

**Table 2:** Descriptive statistics, reliability coefficients, and correlations among the study variables.

Variable	M	SD	α	CR	AVE	1	2	3	4
1. Teacher-regulated generative AI support	3.67	0.71	0.89	0.90	0.64	0.80			
2. Student agency	3.74	0.66	0.87	0.88	0.60	0.52	0.77		
3. Perceived fairness	3.58	0.73	0.88	0.89	0.62	0.48	0.46	0.79	
4. Perceived learning gains	3.81	0.68	0.91	0.92	0.66	0.55	0.63	0.50	0.81

The square roots of AVE are reported on the diagonal. Correlations are reported below the diagonal. All inter-construct correlations were positive and statistically significant at p < 0.001.

α, Cronbach's alpha; CR, composite reliability; AVE, average variance extracted.

All constructs demonstrated satisfactory internal consistency and convergent validity. Cronbach's alpha coefficients ranged from 0.87 to 0.91, composite reliability values ranged from 0.88 to 0.92, and average variance extracted values ranged from 0.60 to 0.66. These values exceeded conventional thresholds for acceptable reliability and convergent validity, indicating that the focal constructs were measured with adequate internal consistency and shared variance.

The correlation analysis showed that all focal variables were positively associated with one another. Teacher-regulated generative AI support was positively correlated with student agency (*r* = 0.52), perceived fairness (*r* = 0.48), and perceived learning gains (*r* = 0.55). Student agency was positively correlated with perceived fairness (*r* = 0.46) and perceived learning gains (*r* = 0.63). Perceived fairness was also positively correlated with perceived learning gains (*r* = 0.50). Among these associations, the strongest correlation was observed between student agency and perceived learning gains, whereas the weakest was observed between student agency and perceived fairness.

In addition, the square roots of the AVE values were greater than the inter-construct correlations, providing evidence of acceptable discriminant validity. Taken together, these descriptive and correlational results provided initial support for the proposed associations among the focal variables and justified proceeding to the measurement and structural model analyses.

### 4.3 Measurement model

Table 3 presents the confirmatory factor analysis results for the measurement model. The proposed four-factor measurement model demonstrated a good fit to the data, χ^2^(113) = 246.38, χ^2^/df = 2.18, CFI = 0.95, TLI = 0.94, RMSEA = 0.053, and SRMR = 0.041. These indices indicate that the measurement model was acceptable and that the four latent constructs were empirically distinguishable. For transparency, the full item wording is reported in Supplementary Table S2. Alternative measurement model comparisons are reported in Supplementary Table S3, and HTMT values are reported in Supplementary Table S4. In addition, the proposed four-factor model fit the data substantially better than alternative three-factor, two-factor, and one-factor models. The three-factor model showed a poorer fit, χ^2^ (116) = 418.52, χ^2^/df = 3.61, CFI = 0.89, TLI = 0.87, RMSEA = 0.078, and SRMR = 0.067. The two-factor model showed an even poorer fit, χ^2^(118) = 671.84, χ^2^/df = 5.69, CFI = 0.81, TLI = 0.78, RMSEA = 0.104, and SRMR = 0.091. The one-factor model demonstrated the weakest fit, χ^2^(119) = 1,052.63, χ^2^/df = 8.85, CFI = 0.68, TLI = 0.63, RMSEA = 0.135, and SRMR = 0.121. These comparisons further support the distinctiveness of the four focal constructs.

**Table 3:** Confirmatory factor analysis results for the measurement model.

Panel A. Model fit indices							
Model	χ^2^df		χ^2^df	CFI	TLI	RMSEA	SRMR
Four-factor measurement model	246.38	113	2.18	0.95	0.94	0.053	0.041
Panel B. Construct-level measurement quality				
Construct	Number of items	Standardized factor loading range	CR	AVE
Teacher-regulated generative AI support	4	0.73–0.84	0.90	0.64
Student agency	4	0.71–0.82	0.88	0.60
Perceived fairness	4	0.74–0.85	0.89	0.62
Perceived learning gains	4	0.76–0.87	0.92	0.66

CFI, comparative fit index; TLI, Tucker–Lewis index; RMSEA, root mean square error of approximation; SRMR, standardized root mean square residual; CR, composite reliability; AVE, average variance extracted.

At the construct level, all four latent variables showed adequate measurement quality. The standardized factor loadings ranged from 0.71 to 0.87 across the four constructs, with no loading falling below the conventional threshold of 0.70. Specifically, the factor loading ranges were 0.73–0.84 for teacher-regulated generative AI support, 0.71–0.82 for student agency, 0.74–0.85 for perceived fairness, and 0.76–0.87 for perceived learning gains. These results suggest that the retained items contributed meaningfully to their intended constructs.

Together with the reliability and AVE results reported above, the CFA findings provide convergent support for the adequacy of the four-factor measurement model. Overall, the fit indices, standardized factor loadings, and construct-level quality indicators support the distinctiveness and adequacy of the measurement model. Consistent with this conclusion, all HTMT values were below the conservative threshold of 0.85, ranging from 0.58 to 0.79.

### 4.4 Structural model and hypothesis testing

Table 4 presents the structural model results, manifest interaction effect, and hypothesis testing results. Overall, the results supported the proposed pattern of direct, indirect, and moderating associations among the focal variables. The structural model also demonstrated an acceptable fit to the data, χ^2^(115) = 252.41, χ^2^/df = 2.20, CFI = 0.95, TLI = 0.94, RMSEA = 0.053, and SRMR = 0.043. In addition, the model explained 31% of the variance in student agency and 42% of the variance in perceived learning gains.

**Table 4:** Structural model estimates, manifest interaction term, and hypothesis testing.

Panel A. Main effects and interaction effect						
Hypothesis	Path	β	SE	*z*	*p*	Result
H1	Teacher-regulated generative AI support → Perceived learning gains	0.24	0.05	4.80	< 0.001	Supported
H2	Teacher-regulated generative AI support → Student agency	0.39	0.06	6.50	< 0.001	Supported
H3	Student agency → Perceived learning gains	0.47	0.06	7.83	< 0.001	Supported
H5	Interaction term of teacher-regulated generative AI support and perceived fairness → Student agency	0.12	0.04	3.00	0.003	Supported
Panel B. Indirect effect test					
Hypothesis	Indirect effect	Effect	Boot SE	95% CI	Result
H4	Teacher-regulated generative AI support → Student agency → Perceived learning gains	0.18	0.04	[0.11, 0.26]	Supported

β, standardized coefficient; SE, standard error; CI, confidence interval. The indirect effect was estimated using bias-corrected bootstrapping with 5,000 resamples. H4 was supported because the 95% confidence interval for the indirect effect did not include zero. The H5 interaction effect was tested using a manifest interaction approach based on the interaction term created from the mean-centered composite scores of teacher-regulated generative AI support and perceived fairness.

#### 4.4.1 Direct effects

Teacher-regulated generative AI support was positively associated with perceived learning gains (β = 0.24, SE = 0.05, *z* = 4.80, *p* < 0.001), supporting H1. Teacher-regulated generative AI support was also positively associated with student agency (β = 0.39, SE = 0.06, *z* = 6.50, *p* < 0.001), supporting H2. In addition, student agency was positively associated with perceived learning gains (β = 0.47, SE = 0.06, *z* = 7.83, *p* < 0.001), supporting H3.

Taken together, these results indicate that teacher-regulated generative AI support was positively associated with both stronger student agency and stronger perceived learning gains, and that student agency was itself positively associated with perceived learning gains.

#### 4.4.2 Mediating effect of student agency

To test the mediating role of student agency, the indirect effect of teacher-regulated generative AI support on perceived learning gains through student agency was examined using bias-corrected bootstrapping procedures. As shown in Table 4, the indirect effect was significant (effect = 0.18, Boot SE = 0.04, 95% CI [0.11, 0.26]), supporting H4. Because the confidence interval did not include zero, the indirect path from teacher-regulated generative AI support to perceived learning gains through student agency was statistically consistent with the proposed mediated pathway.

Because the direct association between teacher-regulated generative AI support and perceived learning gains remained significant after student agency was included in the model, the results indicate a pattern of partial mediation. In other words, teacher-regulated generative AI support was positively associated with perceived learning gains both directly and indirectly through student agency. To further test the conditional nature of the indirect pathway, we estimated the indirect effect at low and high levels of perceived fairness. The conditional indirect effect was weaker at low perceived fairness (effect = 0.12, Boot SE = 0.03, 95% CI [0.06, 0.18]) and stronger at high perceived fairness (effect = 0.24, Boot SE = 0.04, 95% CI [0.16, 0.32]). The difference between these indirect effects was 0.12. In addition, the index of moderated mediation was significant (index = 0.06, Boot SE = 0.02, 95% CI [0.02, 0.10]), indicating that the indirect association between teacher-regulated generative AI support and perceived learning gains through student agency varied significantly across levels of perceived fairness.

#### 4.4.3 Moderating effect of perceived fairness

The moderating effect of perceived fairness on the relationship between teacher-regulated generative AI support and student agency was also significant. Using the manifest interaction approach described above, the interaction term based on the mean-centered composite scores of teacher-regulated generative AI support and perceived fairness significantly predicted student agency (β = 0.12, SE = 0.04, *z* = 3.00, *p* = 0.003), supporting H5. This result indicates that perceived fairness was associated with a stronger positive relationship between teacher-regulated generative AI support and student agency in the proposed model. At the same time, the standardized interaction coefficient was modest in magnitude. Because a separate hierarchical incremental-variance estimate was not computed in the original SEM specification, the substantive interpretation of the moderation effect is based on the standardized interaction coefficient, simple slopes, and conditional indirect effects rather than on an additional ΔR^2^ estimate.

**Figure 2:**
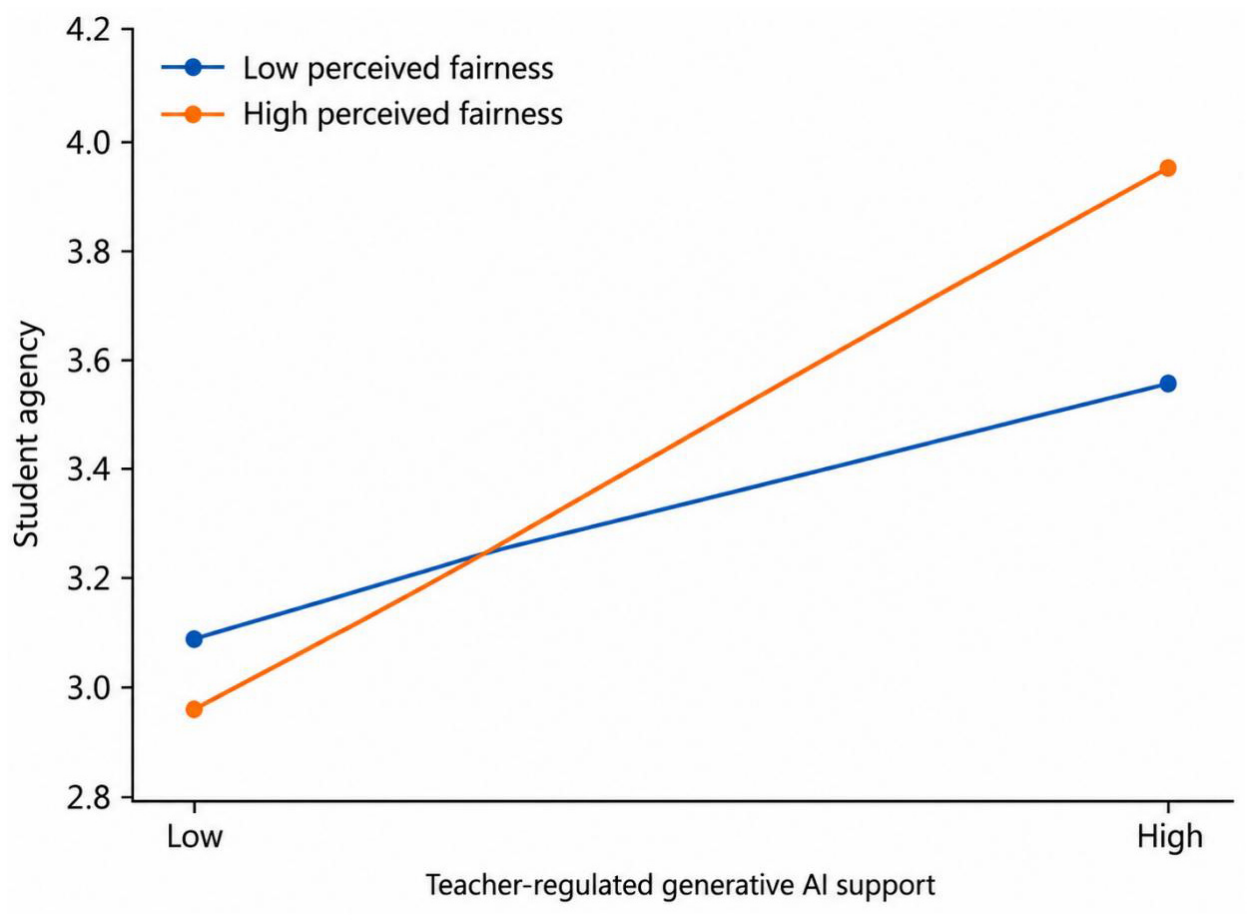
Simple slope plot for the interaction between teacher-regulated generative AI support and perceived fairness in relation to student agency. The interaction was tested using a manifest interaction approach based on the interaction term created from the mean-centered composite scores of teacher-regulated generative AI support and perceived fairness. Values represent predicted levels of student agency at low and high levels of teacher-regulated generative AI support.

To facilitate interpretation, simple slope analyses were conducted at high and low levels of perceived fairness. Figure 2 presents the simple slope pattern for the moderating effect of perceived fairness on the relationship between teacher-regulated generative AI support and student agency. The positive association between teacher-regulated generative AI support and student agency was stronger at high levels of perceived fairness (simple slope = 0.51, *p* < 0.001) than at low levels of perceived fairness (simple slope = 0.27, *p* < 0.001). Thus, the interaction pattern indicated that students were more likely to report stronger agency in response to teacher-regulated generative AI support when they perceived the AI-supported learning environment as fair and transparent.

#### 4.4.4 Summary of hypothesis testing

Overall, the empirical findings supported all proposed hypotheses. Teacher-regulated generative AI support was positively associated with perceived learning gains both directly and indirectly through student agency, and the association between teacher-regulated generative AI support and student agency was stronger when perceived fairness was high. Because the study relied on cross-sectional self-report data, these findings should be interpreted as evidence of a theoretically coherent pattern of associations rather than a confirmed causal pathway. The model accounted for a moderate proportion of variance in the two endogenous variables, explaining 31% of the variance in student agency and 42% of the variance in perceived learning gains. The focal structural paths remained substantively unchanged after including gender, academic level, disciplinary area, and frequency of generative AI use as control variables. Detailed control-variable effects and conditional indirect effects are reported in Supplementary Table S5.

## 5 Discussion

### 5.1 Main findings and overall interpretation

This study examined how teacher-regulated generative AI support is associated with university students' perceived learning gains and whether student agency and perceived fairness help explain this relationship. Three findings are especially relevant. First, teacher-regulated generative AI support was positively associated with perceived learning gains. Second, student agency was statistically consistent with the proposed indirect pathway, suggesting that teacher-guided AI use was related to perceived learning benefits partly because students reported more active ownership and regulation of AI-supported tasks. Third, perceived fairness moderated the association between teacher-regulated generative AI support and student agency, although the magnitude of this interaction was modest.

These findings should be read as evidence of a theoretically coherent pattern of associations rather than as proof of causal effects. They nevertheless point to a useful interpretation of generative AI use in higher education. The educational value of generative AI appears to depend not only on whether students can access AI tools, but also on how those tools are embedded in instructional settings, how students respond to that guidance, and whether the surrounding learning conditions are experienced as fair. This reading is consistent with human-centered accounts of AI and adds a more specific learning-process explanation by locating student agency between teacher-guided AI use and perceived learning gains (Shneiderman, 2022; Xia et al., 2025).

### 5.2 Teacher-regulated AI support, student agency, and socio-technical learning systems

A key theoretical implication of the study is that teacher-regulated generative AI support should not be understood simply as another form of tool access. From a socio-technical perspective, technology use is shaped by the interaction between technical artifacts, users, rules, norms, and organizational practices (Orlikowski, 2000). In the present context, generative AI becomes part of a learning system in which teachers define expectations, students make decisions about use, and institutional or classroom rules shape what counts as legitimate AI-supported work. Teacher regulation therefore functions less as a restriction on technology and more as a local governance arrangement that organizes how AI is used for learning.

This point also links the model more directly to Information Systems theory. TAM and UTAUT emphasize perceived usefulness, ease of use, social influence, and facilitating conditions as important factors in technology acceptance and use (Davis, 1989; Venkatesh et al., 2003). Teacher-regulated generative AI support is conceptually close to facilitating conditions because it provides students with guidance, task structure, and acceptable-use boundaries. However, the present findings suggest that in educational settings, facilitating conditions are not only technical or organizational supports; they also have pedagogical content. They shape whether students use AI reflectively, critically, and with a sense of responsibility for their own learning.

The mediating role of student agency is therefore central. Generative AI can support learning by providing explanations, drafts, examples, and feedback, but these affordances do not automatically produce substantive learning. Students still need to judge the relevance of AI-generated output, revise it in relation to task demands, and decide when to rely on or reject AI suggestions. Here, agency is not only an individual trait that students bring to technology use; it is also a process that can be supported or weakened by the instructional environment. Teacher regulation may support agency when it provides enough structure to reduce uncertainty while still leaving students responsible for evaluating and improving their work.

The result also points to a cautious implication. If teacher regulation becomes overly prescriptive or surveillance-oriented, it may reduce rather than support agency. The positive association found in this study should therefore not be interpreted as evidence that more control is always better. Rather, the findings suggest that pedagogical regulation may be beneficial when it clarifies goals, boundaries, and revision expectations without removing students' responsibility for judgment and self-direction. This tension between structure and autonomy is important for future research on human-centered AI-supported learning.

### 5.3 Perceived fairness and the modest moderation effect

The moderation finding adds a contextual layer to the model. Students reported stronger agency in response to teacher-regulated AI support when they perceived the AI-supported learning environment as fair. This suggests that fairness perceptions may influence how students interpret teacher guidance. When AI-related rules, opportunities, and assessment standards are seen as transparent and consistently applied, teacher regulation is more likely to be experienced as legitimate support. When they are seen as unclear or unequal, even well-intended guidance may be interpreted as arbitrary or restrictive.

The moderation effect was also modest in magnitude (β = 0.12). This point is important. The interaction should not be read as evidence that perceived fairness is a dominant driver of student agency or perceived learning gains. Rather, fairness appears to provide a modest contextual enhancement of the association between teacher-regulated AI support and student agency. Because a separate hierarchical incremental-variance estimate was not computed, the substantive interpretation of the moderation effect is based on the standardized interaction coefficient, simple slopes, and conditional indirect effects. The finding therefore supports the view that fairness is a meaningful but not overriding boundary condition.

This cautious reading is theoretically useful. It suggests that fairness does not replace pedagogical regulation or learner agency as the central focus of the model. Instead, fairness helps explain when students may be more willing to take up teacher-guided AI use actively. This interpretation also connects educational AI research with governance-oriented discussions of transparency, equity, and legitimacy (Coman et al., 2026; García-López and Trujillo-Liñán, 2025; UNESCO, 2023). Fairness is therefore not only an external ethical concern; it is also part of the perceived learning context within which AI-supported pedagogy is enacted.

There are also possible alternative explanations. Students who are generally satisfied with a course may evaluate both teacher regulation and fairness more positively. Similarly, students who perceive stronger learning gains may retrospectively judge the AI-supported learning environment as more legitimate. These possibilities cannot be ruled out with cross-sectional self-report data. They suggest that fairness should be examined in future studies using longitudinal, experimental, or multi-source designs that can separate perceived fairness from related constructs such as trust, satisfaction, and institutional policy clarity.

### 5.4 Theoretical implications

The study contributes to research on generative AI in higher education in an incremental but meaningful way. It does not claim that previous research has ignored AI-supported pedagogy, AI literacy, instructional design, or governance. Instead, it shows how these concerns can be connected in a single empirical model by linking pedagogical regulation, learner agency, and fairness. This reframing helps move the discussion from broad claims about AI access toward a more relational account of how AI-supported learning is organized and experienced.

The study also contributes to Information Systems-oriented discussions of technology-enabled learning. TAM and UTAUT are useful for understanding technology acceptance, perceived usefulness, ease of use, social influence, and facilitating conditions (Davis, 1989; Venkatesh et al., 2003). The present study complements these frameworks by showing that, in AI-supported learning, facilitating conditions may take a specifically pedagogical form. Teacher-regulated generative AI support provides guidance, boundaries, and interpretive expectations that may help students use the technology more agentically. Thus, the study extends rather than replaces IS adoption perspectives by bringing learner agency and pedagogical regulation into the analysis of generative AI-supported education.

A further theoretical contribution concerns fairness. Existing discussions often treat AI fairness primarily as a governance or ethical issue. The present findings suggest that perceived fairness also has learning-process relevance because it conditions the association between teacher regulation and student agency. This does not mean that fairness has a large independent effect in the present data; the moderation effect was modest. However, it does suggest that students' perceptions of transparency, consistency, and equity may influence whether teacher guidance is experienced as legitimate and worth actively taking up. This provides a bridge between responsible AI governance and human-centered learning design.

Finally, the findings highlight a tension that deserves more theoretical attention. Generative AI can increase student autonomy by providing flexible support, but it can also increase dependence if students rely on generated output without judgment. Teacher regulation can support agency by providing structure, but it can also constrain agency if it becomes too controlling. The value of AI-supported learning may therefore depend on finding a productive balance between guidance and autonomy, efficiency and reflection, and technological support and human judgment.

### 5.5 Practical implications

For higher education institutions, the findings point beyond a simple permission-or-prohibition approach to generative AI. Students may benefit more when teachers explain what kinds of AI use are appropriate for a task, clarify acceptable-use boundaries, and require students to evaluate and revise AI-generated output rather than adopt it directly. Here, teacher regulation should not be understood only as restriction. It can also be a pedagogical arrangement that helps students use AI more responsibly and reflectively.

Instructors can also design AI-supported assignments that preserve student agency. For example, students can be asked to compare several AI-generated responses, identify errors or limitations, justify their final choices, and explain how their own work changed after using AI. Such tasks make AI use visible without removing students' responsibility for learning. This is especially important because the value of AI-supported learning depends not only on the availability of AI-generated support, but also on whether students remain active evaluators and authors of their work.

The results further suggest that fairness conditions should be made explicit. Teachers and institutions should communicate AI-use rules clearly, apply assessment standards consistently, and avoid situations in which some students have substantially better AI-supported learning opportunities than others. Because the moderation effect in this study was modest, fairness should not be treated as a single solution to AI-supported learning challenges. Nevertheless, transparent and equitable conditions may help students perceive teacher-guided AI use as legitimate and may increase their willingness to engage with it actively.

### 5.6 Limitations and future research

Several limitations should be acknowledged when interpreting the findings of this study. First, the study relied on cross-sectional self-report data, which limits causal inference. Although the proposed model was theoretically grounded and the structural results were internally coherent, the findings should be interpreted as relational rather than strictly causal. Reverse causality and reciprocal interpretation remain possible. Future research could use longitudinal, experimental, or classroom intervention designs to examine how teacher-regulated generative AI support, student agency, and perceived fairness develop over time and whether changes in these variables are associated with subsequent learning outcomes.

Second, the study focused on perceived learning gains rather than objective performance outcomes. Perceived gains are important because they capture students' experienced sense of progress, understanding, and academic benefit, yet they do not fully substitute for direct indicators of achievement, transfer, or task quality. Future studies could therefore combine perceptual measures with performance-based indicators, such as assignment quality, revision quality, course achievement, behavioral trace data, or teacher-rated performance, in order to provide a more comprehensive account of the educational value of generative AI in higher education.

Third, the study was conducted with university students from six institutions in western China and relied on convenience sampling. Although this context provides a meaningful setting for examining authentic AI-supported learning experiences, the generalizability of the findings to other regions, disciplinary contexts, or educational levels remains uncertain. Future research could test the proposed model across different institutional and cultural settings and compare whether the roles of pedagogical regulation, learner agency, and perceived fairness vary across disciplines, course types, or national policy environments.

Fourth, teacher-regulated generative AI support was examined at the level of student perception. While this is appropriate for understanding learners' experiences, it cannot fully capture how pedagogical regulation is enacted in practice. In addition, the recalled learning episodes may have involved heterogeneous courses, tasks, and AI tools, which introduces further variability into the study context. Future studies could strengthen the evidence base by combining student reports with teacher reports, classroom observations, instructional materials, prompt-use records, or trace data on actual AI-supported learning processes. Such multi-source approaches would make it possible to distinguish more clearly between intended teacher regulation, perceived regulation, and enacted regulation in classroom contexts.

Fifth, the two context-specific measures introduced in this study should be interpreted as initial operationalizations rather than fully validated standardized scales. The study used expert review, pilot testing, CFA, CR, AVE, HTMT, and alternative measurement model comparisons, but it did not calculate a formal CVI or conduct EFA before CFA. Future research should further examine content validity, factor structure, measurement invariance, predictive validity, and cross-sample stability of teacher-regulated generative AI support and perceived fairness in AI-supported learning.

Finally, future research could examine additional explanatory pathways and contextual factors that may shape the relationship between pedagogically regulated AI support and learning outcomes. Because all focal variables were assessed through the same questionnaire, common method inflation cannot be fully ruled out (Podsakoff et al., 2003; Conway and Lance, 2010). In addition, the present study tested moderation using a manifest interaction rather than a latent interaction approach, and the model did not include teacher-side or course-level variables. Future research could therefore examine AI literacy, academic integrity beliefs, task complexity, disciplinary norms, assessment design, and course-level pedagogical features as additional influences on how students interpret and respond to teacher-regulated generative AI support. Exploring these factors would deepen understanding of when and how teacher-regulated generative AI support is most likely to be associated with perceived learning gains in higher education.

## 6 Conclusion

This study examined how teacher-regulated generative AI support relates to university students' perceived learning gains in higher education, with student agency as a mediator and perceived fairness as a moderator. The findings showed that teacher-regulated generative AI support was positively associated with perceived learning gains both directly and indirectly through student agency. In addition, perceived fairness was associated with a stronger positive relationship between teacher-regulated generative AI support and student agency.

These findings suggest that the educational value of generative AI in higher education is not explained by tool access alone, but by how AI use is pedagogically regulated, actively taken up by learners, and experienced under fair learning conditions. More broadly, the study contributes to current discussions of human-centered and responsible AI integration in higher education by clarifying one pathway through which teacher-regulated generative AI support may be associated with perceived learning gains. Because the evidence is based on cross-sectional self-report data and concerns perceived rather than objective learning outcomes, the conclusions should be interpreted with appropriate caution. Taken together, these findings suggest that meaningful AI integration in higher education is more likely when instructional designs help students remain active, reflective, and responsible participants in their own learning.

## Data Availability

The raw data supporting the conclusions of this article will be made available by the authors, without undue reservation.
