# Supplementary material for: Teacher-regulated generative AI support, student agency, and perceived learning gains in higher education: the moderating role of perceived fairness
Source: Front Artif Intell. 2026 Jul 13;9:1863666. doi: 10.3389/frai.2026.1863666 (PMC13402406; doi:10.3389/frai.2026.1863666)
Supplement: Supplementary file 1 [file Supplementary_File_1.pdf]

Supplementary Material Tables S1-S5

Table S1. Construct sources, adaptation, status, and item focus.

Construct	Sources	Adaptation	Status	Item focus
Teacher-regulated generative AI support	Teacher roles in GenAI-supported higher education and AI-augmented teaching design (Chan and Tsi, 2024; Niu et al., 2026)	Items were composed to capture AI-specific pedagogical regulation, including goal framing, acceptable-use boundaries, interpretive guidance, and revision expectations.	Context-specific items newly developed from prior conceptual literature	Clear explanation of AI use, guidance on interpreting outputs, and expectations for revision after AI use
Student agency	Agentic engagement, learning agency, and agentic AI in higher education (Reeve, 2013; Xia et al., 2025; Alqurni, 2026)	Items were adapted to reflect active decision-making, evaluation of AI output, revision, and ownership of the learning process in teacher-guided GenAI use.	Adapted and contextually revised	Purposeful AI use, critical evaluation of AI suggestions, and maintaining ownership of final work
Perceived fairness	Transparency, equity, and governance in educational AI (García-López and Trujillo-Liñán, 2025; UNESCO, 2023; Coman et al., 2026)	Items were composed to reflect student perceptions of transparent rules, equitable access to AI-supported opportunities, and consistency in evaluation standards.	Context-specific items newly developed from prior conceptual literature	Clear and transparent AI-use rules, fair opportunities to benefit from AI, and consistent teacher evaluation standards
Perceived learning gains	Perceived academic learning impact of AI use in higher education (Dogaru et al., 2025; Thomson et al., 2024)	Items were adapted to capture perceived improvement in understanding, work quality, efficiency, and perceived academic progress under teacher-guided GenAI use.	Adapted and contextually revised	Better understanding of course content, improved academic work quality, and more efficient learning progress

Note. The table summarizes construct sources, adaptation, item-development status, and representative item focus. All items were reviewed by three experts and piloted with 40 students before full administration. Full item wording is reported in Supplementary Table S2.

Table S2. Full item wording, source basis, and adaptation status.

Construct	Item wording	Source basis	Adaptation status
Teacher-regulated generative AI support	1. Teachers clearly explained how generative AI should be used in course learning.<br>2. Teachers provided guidance on how to evaluate or interpret AI-generated content.<br>3. Teachers set clear expectations for revising or improving work after using generative AI.<br>4. Teachers helped students use generative AI in ways that supported learning rather than simple task completion.	Chan and Tsi (2024); Niu et al. (2026)	Context-specific items newly developed from prior conceptual literature
Student agency	1. I actively decided how to use generative AI to support my learning.<br>2. I evaluated whether AI-generated suggestions were appropriate for my task.<br>3. I revised my work based on my own judgment rather than relying entirely on AI output.<br>4. I used generative AI in ways that helped me take greater control of my learning process.	Reeve (2013); Xia et al. (2025); Alqurni (2026)	Adapted and contextually revised
Perceived fairness	1. The rules for using generative AI in this course were clear and transparent.<br>2. Students had fair opportunities to benefit from generative AI support.<br>3. The teacher applied similar standards when evaluating AI-supported work.<br>4. I felt that the use of generative AI in this learning context was managed fairly.	García-López and Trujillo-Liñán (2025); UNESCO (2023); Coman et al. (2026)	Context-specific items newly developed from prior conceptual literature
Perceived learning gains	1. Using generative AI in this course helped me better understand the learning content.<br>2. Generative AI support helped me improve the quality of my academic work.<br>3. I learned more efficiently when generative AI was used under teacher guidance.<br>4. My use of generative AI in this learning context contributed to meaningful learning progress.	Dogaru et al. (2025); Thomson et al. (2024)	Adapted and contextually revised

Note. Table S2 reports the full item wording used in the questionnaire together with the source basis and adaptation status of each construct. Supplementary Tables S3-S5 report alternative measurement model comparisons, HTMT values, and additional structural estimates.

Table S3. Alternative measurement model comparisons.

Model	χ^2	df	χ^2/df	CFI	TLI	RMSEA	SRMR
Four-factor model	246.38	113	2.18	.95	.94	.053	.041
Three-factor model	418.52	116	3.61	.89	.87	.078	.067
Two-factor model	671.84	118	5.69	.81	.78	.104	.091
One-factor model	1052.63	119	8.85	.68	.63	.135	.121

Note. The proposed four-factor model fit the data substantially better than the alternative three-factor, two-factor, and one-factor models, supporting the distinctiveness of the focal constructs.

Table S4. HTMT values among the focal constructs.

Construct	1	2	3	4
1. Teacher-regulated generative AI support	-			
2. Student agency	.68	-		
3. Perceived fairness	.61	.58	-	
4. Perceived learning gains	.70	.79	.64	-

Note. All HTMT values were below the conservative threshold of .85, providing additional evidence of discriminant validity.

Table S5. Control-variable effects and conditional indirect effects.

Analysis	Path/Effect	Estimate	SE	p / 95% CI
Control effect	Gender → Student agency	.04	.04	.372
Control effect	Academic level → Student agency	.09	.05	.048
Control effect	Disciplinary area → Student agency	.06	.04	.181
Control effect	Frequency of generative AI use → Student agency	.16	.05	.002
Control effect	Gender → Perceived learning gains	.03	.04	.511
Control effect	Academic level → Perceived learning gains	.05	.05	.284
Control effect	Disciplinary area → Perceived learning gains	.07	.04	.146
Control effect	Frequency of generative AI use → Perceived learning gains	.11	.05	.021
Conditional indirect	Low perceived fairness	.12	.03	[.06, .18]
Conditional indirect	High perceived fairness	.24	.04	[.16, .32]
Moderated mediation	Index of moderated mediation	.06	.02	[.02, .10]

Note. Estimates for the conditional indirect effects and the index of moderated mediation are based on bias-corrected bootstrapping. The focal structural paths remained substantively unchanged after the control variables were included.
